# Infectious Bronchitis Virus Evolution, Diagnosis and Control

**DOI:** 10.3390/vetsci7020079

**Published:** 2020-06-22

**Authors:** Matteo Legnardi, Claudia Maria Tucciarone, Giovanni Franzo, Mattia Cecchinato

**Affiliations:** Department of Animal Medicine, Production and Health, University of Padua, Viale dell’Università, 16, 35020 Legnaro, Italy; matteo.legnardi@phd.unipd.it (M.L.); giovanni.franzo@unipd.it (G.F.); mattia.cecchinato@unipd.it (M.C.)

**Keywords:** infectious bronchitis virus, coronavirus, viral evolution, diagnosis, control, vaccination

## Abstract

RNA viruses are characterized by high mutation and recombination rates, which allow a rapid adaptation to new environments. Most of the emerging diseases and host jumps are therefore sustained by these viruses. Rapid evolution may also hinder the understanding of molecular epidemiology, affect the sensitivity of diagnostic assays, limit the vaccine efficacy and favor episodes of immune escape, thus significantly complicating the control of even well-known pathogens. The history of infectious bronchitis virus (IBV) fits well with the above-mentioned scenario. Despite being known since the 1930s, it still represents one of the main causes of disease and economic losses for the poultry industry. A plethora of strategies have been developed and applied over time, with variable success, to limit its impact. However, they have rarely been evaluated objectively and on an adequate scale. Therefore, the actual advantages and disadvantages of IBV detection and control strategies, as well as their implementation, still largely depend on individual sensibility. The present manuscript aims to review the main features of IBV biology and evolution, focusing on their relevance and potential applications in terms of diagnosis and control.

## 1. Infectious Bronchitis Virus

Infectious bronchitis virus (IBV) is currently classified within the species *Avian coronavirus*, genus *Gammacoronavirus*, family *Coronaviridae*, order *Nidovirales.* IBV is an enveloped virus with a single-stranded positive-sense RNA genome of approximatively 27 kb, which displays the following gene organization: 5′UTR-1a/1ab-S-3a-3b-E-M-5a-5b-N-3′UTR. The RNA molecule has a 5′ *cap* and a 3′ poly(A) tail and acts as mRNA for translation of the replicase polyproteins, whose coding regions occupy the 5′ two-thirds of the viral genome. The two untranslated regions (UTR) play a key role in virus replication interacting with viral replicases and potentially other host proteins [1,2]. Non-structural proteins (nsp) are encoded by two open reading frames (ORFs) (ORF1a and ORF1ab), which are translated in 2 polyproteins (pp1a and pp1ab) through a -1 ribosomal frameshift. The polyproteins are then cleaved into 15 nsps (nsp2-16) by autoproteolytic activity. The function of each protein is reviewed in Jackwood et al. [3].

The Spike (S) protein is the most described IBV structural protein. This transmembrane protein, organized in trimers, determines the typical crown-like aspect of coronaviruses, observable at electron microscopy [4]. It is post-translationally cleaved in 2 sub-units, S1 and S2. The S1 contains at least 2 domains potentially involved in host receptor binding [5] and is therefore considered the main determinant of host and tissue tropism [4,6]. For the same reason, the most relevant epitopes are located on this protein, including neutralizing ones. The high intensity of the selective pressures driven by the host immune response acting on this region is probably responsible for its remarkable genetic heterogeneity, both among coronaviruses and within IBV [3,5,7]. Because of strains heterogeneity and linkage with biological features, the Spike-coding gene has been used to classify IBV variants into genotypes for a long time. However, the absence of common classification and nomenclature criteria complicated the understanding of IBV molecular epidemiology until recent years [8]. The more conserved S2 sub-unit contains the transmembrane domain anchoring the S protein to the envelope [9] and is fundamental for viral fusion [10]. The interplay between S1 and S2 subunits might determine the overall avidity and specificity of virus attachment and thus viral tissue and host range [11].

The other surface proteins, Membrane (M) and Envelope (E), are involved in virus morphogenesis and assembly. Besides E and S, the M protein interacts with the Nucleocapsid (N) one, which directly binds genomic RNA to form a helical ribonucleoprotein complex and probably participate in RNA packaging [12,13].

## 2. Viral Evolution

Mutation is the ultimate source of genetic polymorphism and represents the first fundamental substratum for evolution, since it generates the genotypic (and phenotypic) variants that spread and become fixed through genetic drift or natural selection [14,15,16]. 

RNA viruses are known for having an extremely high error rate (approximately 10^−4^ to 10^−5^ misincorporations per nucleotide position) because of the lack of RNA-dependent RNA polymerase (RdRp) proofreading activity coupled with the absence of RNA repair mechanisms [17,18]. This leads to approximately 1 mutation∙genome^−1^ replication^−1^, which is a 10-fold higher rate than retroviruses and 10,000-fold greater than most DNA viruses, probably justifying the limited genome size of RNA viruses. In fact, while the continuous production of mutants favors virus adaptability in the event of environmental changes, the accumulation of multiple mutations in the same viral particle would lead to a dramatic fitness reduction [19]. 

Coronaviruses, including IBV, with their 30 kb long genome, can be considered an exception within the RNA virus group since their large replicase gene contains an ExoN domain in the nsp14, related to host proteins of the DEDD superfamily of exonucleases and involved in proofreading and repair activity. Nevertheless, the estimated substitution rate of IBV (10^−4^–10^−5^ substitutions/site/year) is still remarkable [20] and guarantees a noteworthy evolutionary potential. This reflects on the heterogeneity of variants that have emerged all around the world [14,15]. In fact, the current classification, based on the S1 sequencing, includes 6 genotypes, further divided into 32 lineages, exhibiting a pairwise genetic distance of 30% and 13%, respectively [8]. However, the fate of both historical and new variants is largely unpredictable: some circulate at worldwide level for decades, while others show a limited temporal and spatial persistence [14,16]. Experimental evidences revealed the emergence of different mutants even within the same host during infection [21] or within single vaccine lots [22]. Interestingly, vaccines featured by a higher number of subpopulations were associated with higher replication titres within the host and the induction of a stronger immune response, potentially attributable to the replication ability in different tissues and a broader epitopic spectrum [23]. Despite apparently advantageous from certain perspectives, the presence of viral subpopulations can increase the risk of reversion to virulence due to the selection of more fit variants, as suggested by the occurrence of post-vaccine reactions induced by subpopulation-rich vaccines [24,25].

While the random selection of IBV variants (genetic drift) is often implicitly stated in many manuscripts, the actual role of natural selection has been explicitly accounted less frequently. More recently, the relevance of natural selection in shaping IBV evolution has been determined for both vaccine and field strains. In longitudinally monitored birds vaccinated with live-attenuated Ark vaccines, for which subpopulations differed in the S protein sequence, minor viral subpopulations were rapidly selected and mutations emerged during passages in chickens, suggesting that the phenotype of these subpopulations was advantageous and/or favored adaptation to the chicken host [22,24]. Accordingly, when chicken-derived strains were re-isolated and passaged in eggs, at least some of them reverted to the original vaccine sequence in some positions [22]. Minor modifications in the S protein are likely responsible for the different IBV affinity for receptors located in different tissues, thus affecting both tropism and pathogenicity [4,26].

A study by Gallardo et al., based on S sequence analysis, highlighted that the predominant IBV phenotype contained in the vaccine became a minor population in the host, and also significant differences were detected in the incidence of some distinct IBV populations in the considered tissues and fluids. Therefore, in addition to the host role, the specific tissues could exert a certain pressure, selecting the variants able to replicate better in a particular microenvironment [23].

Closely related strains sometimes demonstrate a relevant heterogeneity in cross-neutralization patterns, highlighting that mutations in specific amino-acid sites are responsible for immune evasion [15]. Accordingly, strain D1466 (G-II) and the recently emerged D181 variant, although being formally classified within the same lineage based on the overall S1 genetic identity, demonstrated only 9% cross-relationship [27].

**Figure 1:**
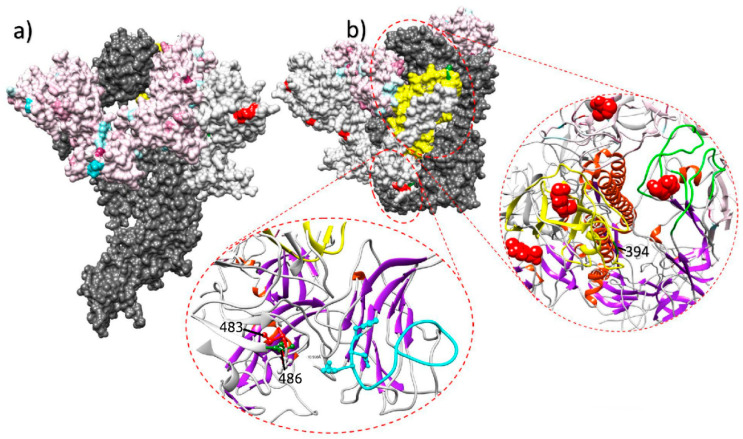
Lateral (**a**) and upper (**b**) view of the quaternary structure of the IBV spike protein. The full spike protein is reported for one monomer only (dark gray), while other monomers have been edited to highlight different selective pressure features. In the color-coded monomer, sites under a stronger selective pressure before the homologous vaccination introduction are reported in light blue, while the ones under a stronger selective pressure after the vaccination change are coded in purple. In the white-colored monomer, sites under episodic diversifying or directional selection in the post-vaccination change period are reported in red and green, respectively. The region demonstrating the stronger diversification tendency after the homologous vaccination introduction is highlighted in yellow. The ribbon visualization of relevant protein domains is reported as inserts. Lower insert reports S1 N-terminal receptor binding motif and the partial ceiling over it (coded in light blue). Sites of the nearby monomer under episodic diversifying and directional selection in the post-vaccination period are reported in red and green, respectively. The right insert displays the S1 C-terminal domain region. Two monomers are represented: one depicting the region with the highest diversification tendency after the homologous vaccination introduction (in yellow) and the other highlighting the corresponding extended putative receptor binding motif loops (in green). Figure from [7].

Immune response is thus expected to be the main selective force acting on IBV evolution, at least in the antigenic regions. Some experimental evidences seem to further support this hypothesis. When field strains were inoculated in vaccinated and unvaccinated chicken groups, the emergence of non-synonymous mutations was described only in some of the vaccinated birds [28]. Evidences of vaccine-driven immune selection were also provided by a recent epidemiological study performed in field conditions, where an increase in the selective forces acting on the S1 protein of QX strains has been documented after the introduction and mass application of a homologous vaccine [7]. The diversification tendency after the vaccination strategy change was especially strong on sites exposed on the Spike surface and particularly in or nearby receptor binding motifs, which can be considered likely target of effective immune response (Figure 1).

However, it should be emphasized that vaccination is not “per se” a risk factor for viral evolution, but improper vaccination can be. Natural selection is expected to predominate when Ne∙S >> 1 (i.e., effective population size × selection coefficient is much higher than 1). While vaccination clearly imposes a strong selection coefficient, it also drastically reduces viral replication and therefore the population size, preventing the emergence of more fit variants. 

On the other hand, improper vaccine selection or administration, as well as factors suppressing the bird immune response, can allow the virus to circulate in a partially immune environment, potentially leading to the emergence, selection and spreading of immune escape variants [29,30,31].

Current studies are essentially based on the S protein (or part of it), representative of less than 10% of IBV genome, whereas IBV virulence, pathogenesis and tropism are polygenic and also associated with genetic variants outside of the S gene [32,33,34], especially in, but not limited to, the replicase gene [35,36]. Additionally, most of the currently available studies were based on sequences obtained through Sanger sequencing, which allows to investigate only major subpopulations. Benefits will surely derive from the wider application of Next Generation Sequencing (NGS) technologies to the study of whole-genome IBV dynamics within the host, and by the integration of these data with the evolutionary patterns observed at the epidemiological level. In fact, the relationship between the within-individual and population level fitness is still unknown, as well as the reasons causing some variants to remain restricted to limited geographic areas and time periods, while others are able to persist and spread worldwide.

A second pivotal source of genetic variability is recombination. Coronaviruses are featured by a high recombination rate, mediated by a copy-choice mechanism [37]. In this process, the RdRP switches from one RNA molecule (the donor template) to another (the acceptor template) during the synthesis while bound to the nascent nucleic acid chain, generating a chimeric RNA molecule with mixed ancestry. The strategy adopted by coronaviruses in gene expression regulation facilitates this phenomenon since it depends on discontinuous transcription, which relies entirely on the viral RdRp template-switching property [13,38]. IBV makes no exception: several studies reported recombination events which occurred in field conditions, involving both field and vaccine strains [39,40,41]. This evidence has risen some concerns on the potential risk of new recombinant strain emergence, especially when multiple vaccines are administered and/or vaccine strains circulate for a long time. Single detections or small clusters of recombinant strains are typically reported, suggesting the limited fitness of most strains. However, some noteworthy exceptions have been described. A study performed in Italy and Spain reported 4 different recombination events involving QX and 793B strains [42]. While Italian recombinants were only sporadically detected, the Spanish ones emerged among the dominant field strains in the region. Therefore, both positive and negative selection can act on recombinant viruses, likely depending on the peculiar recombination pattern and local epidemiological context. 

Besides affecting viral biology, recombination represents a remarkable challenge for IBV classification. As previously mentioned, the current classification is officially based on the S1 gene sequencing [8], even though the hypervariable regions of this segment are usually sequenced because of practical and economic constraints. Little is known about the rest of IBV genetic backbone, where frequent recombination events involving even distantly related strains are likely [43,44,45]. Therefore, the S1-based classification could be poorly representative of the actual IBV evolutionary history and biological properties. Additionally, recombination analysis is rarely performed, leading to the risk of misclassifying chimeric viruses as new genotypes.

## 3. Approaches to IBV Diagnosis

While zootechnical parameters and presence of symptoms are useful as preliminary evidences of infectious bronchitis (IB) outbreaks, IBV-induced signs are not pathognomonic. Laboratory assays are therefore mandatory to detect and characterize IBV strains. The most used diagnostic tests include viral isolation, serological and molecular assays. 

### 3.1. Viral Isolation

When attempting to isolate field viruses, samples should be promptly collected when IB-compatible signs are at first observed, since IBV titres peak in the first week post-infection, possibly before clinical manifestations [46]. Tracheal tissues or swabs are the sample of choice especially in the acute phase, but kidneys or oviduct are also suitable sites, especially in presence of lesions. Isolation may also be attempted from caecal tonsils and cloacal swabs, but viral recovery rate seems lower [46]. To investigate IBV presence at flock or farm level, pooled samples should be taken from both symptomatic and healthy animals. Samples should be carefully stored on ice and rapidly sent to the laboratory to preserve virus viability. Allantoic cavity of embryonated eggs or tracheal organ cultures (TOCs) from specific pathogen-free (SPF) chickens are considered more efficient than cell culture [47]. IBV causes urate deposits in mesonephros, stunting, curling and embryonic death when cultured on embryonated eggs, and ciliostasis on TOCs. Lesions are usually observed within the third passage when using embryonated eggs, while ciliostasis on TOCs usually occurs after the first passage [48]. In either case, the presence of these signs does not allow to ascertain IBV presence. Serological or biomolecular methods should be used to confirm and further characterize the isolated strain [49]. Nowadays, viral isolation is not routinely performed for diagnostic purposes because of its lengthiness and stringent requirements, but it is still useful for several other scopes such as vaccine production, sample enrichment before whole genome sequencing, pathogenicity tests and evaluation of the protection conferred by vaccines against virulent challenges. 

### 3.2. Serology

Methods based on antibody detection are used to study previous IBV circulation or assess the induced immunological response. A range of serological tests is available, including agar gel precipitation (AGP), enzyme-linked immunosorbent assay (ELISA), virus neutralization (VN) and hemagglutination inhibition (HI).

AGP allows for quick and inexpensive responses but is not routinely adopted since it lacks in sensitivity and the main precipitating antibodies (i.e., IgM) are detected only for a few weeks after exposure [46].

Commercial ELISA tests are the most commonly applied for routine serological monitoring because of their cost-effectiveness and rapid turnaround. Antibody titration is useful to monitor both the vaccination response, especially in layers and breeders, and exposure to field virus. For a proper interpretation, baseline values should be established based on a regular monitoring of the local situation since antibody titres depend on many factors like breed, type, age at sampling, vaccination program and schedule [50,51]. 

The observed results should be compared to the baseline in terms of average titres, within-group uniformity and persistence. Reasonably high, uniform and lasting titres are indicative of a proper vaccination, while low, uneven and short-lived titres suggest a defective vaccination, likely due to an improper administration or poor quality of the vaccine batch. When the observed titres are significantly higher than expected, a field infection should be suspected.

The majority of the available ELISA kits are designed to detect polyclonal antibodies against the whole virion and do not allow serotypization. Several alternatives to commercially available ELISA kits have been developed [52,53,54,55,56], some of which are based on serotype- or strain-specific monoclonal antibodies [57,58,59], but their use in routine diagnostics is currently limited.

VN and HI tests can identify serotype-specific antibodies and are therefore useful to assess the specific response to both field and vaccine strains. However, only VN should be considered the method of choice for serotyping purposes, because of the more frequent occurrence of cross-reactions using HI [60]. Unfortunately, the adoption of VN for routine monitoring is limited by its laboriousness. 

Aside from the described methods, other serological assays have been recently developed, including methods based on Unknown multi-analyte profiling (xMap) [61], microarray [62] and strip-based [63] technologies, but they are not commercially available yet.

### 3.3. Molecular Techniques

Currently, biomolecular assays are the most used tools for IBV detection, because of their sensitivity and quick response time. Besides viral RNA detection, they allow to characterize the detected strains from a genetic standpoint, allowing to properly plan and evaluate vaccination protocols and assess the presence of specific field strains. It is worth noting that the mere PCR positivity does not imply an active infection at the moment of sampling since genome traces can persist for a relatively long period after viral clearance. Thus, the results should be carefully interpreted. 

A multitude of RT-PCR and qRT-PCR-based methods have been validated, either generic and targeting virtually all IBV subtypes, or genotype- or strain-specific [64,65,66,67,68]. The most commonly targeted region is the S1 gene, where the genetic variability featuring IBV variants is concentrated [3]. Due to the vast adoption of live vaccines, which may persist for the whole production cycle in broilers, the majority of samples usually proves positive to generic assays, and further characterization, either by sequencing or a panel of specific assays, is required to obtain meaningful results. Nevertheless, when multiple strains are present, generic RT-PCR followed by Sanger sequencing would detect only one strain, typically the one with the highest titre. However, the differential primers affinity may also bias the results. For this reason, it is not uncommon for different assays to lead to the detection of different strains [69]. On the other hand, the use of strain-specific tests only would limit the diagnostic sensitivity to an *a priori* subset of IBV strains or genotypes. In fact, the ideal diagnostic algorithm should comprise both generic and specific assays selected on the actually circulating variants in the field. 

Restriction fragment length polymorphism (RFLP) has been used as an alternative to sequencing and specific probes development. The use of restriction enzymes represents a quick, inexpensive technique, but, unfortunately, not all strains can be differentiated in this way [70], and the adopted enzymes would require periodic updates to keep up with IBV evolution.

Quantitative real-time RT-PCR (qRT-PCR) allows the quantification of the targeted genetic material present in the sample. Real-time assays targeting conserved regions of IBV genome cannot quantify the respective contribution of simultaneously present strains, limiting their usefulness to screening purposes. On the other hand, a properly chosen panel of strain-specific qRT-PCR assays enables their precise differentiation. Field strains quantification may help to discriminate an incidental detection from a disease primarily caused by IBV, while vaccine strain titres can be used to assess vaccine coverage, replication and persistence. The description of vaccine kinetics in longitudinal studies may be of great aid in characterizing different vaccination protocols, replication dynamics of the various vaccines and potential interactions when co-administered [68].

Currently, the main issue of PCR-based methods used for IBV characterization is the lack of consensus on the genetic classification method. The results provided by different laboratories may differ because the considered genomic region differs in size or position, or because the same genetic subtype is referred to with different names by different laboratories [15]. As previously mentioned, the recently proposed classification by Valastro et al. [8] effectively solved this issue. However, historical strains are still referred to using the traditional nomenclature sometimes by researchers and more often in the field. Unfortunately, the use of full S1 sequencing is not routinely used for monitoring because of its cost and sensitivity limits [71].

The absence of clear genetic markers also prevents the reliable discrimination between vaccine and field strains [72], although consistent genetic differences were sometimes demonstrated [73,74,75]. Anamnestic data like the presence of symptoms and vaccination schedule should thus be taken into account for more confident deductions.

Suitable specimens for PCR testing include dry swabs, tissues and FTA cards. These are paper substrates containing chemicals that protect nucleic acids and inactivate pathogens with no risk of contamination, requiring less stringent conitions for storage and shipment but possibly entailing a loss in sensitivity [71]. Samples are usually collected at tracheal or cloacal level but, depending on the presence of particular symptoms and lesions, they should also be taken from the kidney or oviduct. Different strains may have a different tropism [76], thus samples collected from different districts may lead to different results. Pooled samples are typically considered an acceptable cost/benefit trade-off to obtain information about the epidemiological status of a group of birds as an epidemiological unit, while individual samples should be analyzed to assess the vaccine coverage or prevalence of a field virus within a flock [68].

Besides being vastly adopted for diagnostic purposes, PCR-based methods followed by sequencing are of great use for research studies of IBV epidemiology. For example, phylodynamic projects allow to investigate the evolutionary history and spreading dynamics of the viral population, providing useful information to evaluate and improve IBV control measures on a large-scale perspective [20,77].

Other molecular techniques, such as loop-mediated isothermal amplification (LAMP) and padlock probes (PLPs) combined with rolling circle amplification (RCA), have been validated for IBV detection [78,79]. Additionally, NGS techniques have recently been used to study IBV [80,81]. This approach, despite being currently too expensive and laborious for routine use, has proven useful for research purposes, particularly because it allows to precisely study the presence of subpopulations within a single sample and contextualize IBV within the respiratory disease complex.

## 4. Diagnostic Approach Selection

No technique can be considered fully conclusive by itself, usually requiring the combination of multiple tests to obtain a complete picture of the infectious status of single animals, flocks or larger populations. Rather than implementing a standard set of assays, the best diagnostic approach should probably be decided on a case-by-case basis to suit the particular demands and peculiarities of the epidemiological context. Different tests have different costs and response times, and they may require different types of samples, which are not always easily collected in certain situations (i.e., samples that require the sacrifice of birds, or whose shipment requires stringent precautions). The purpose of the survey is also crucial to decide which tests to use. For instance, the investigation of a suspected IB outbreak may impose a different approach compared to the assessment of vaccination coverage.

In a typical field situation, the common adoption of multiple live vaccines may complicate the diagnostic process and mask the simultaneous presence of field strains [68,74,82]. For this reason, the implemented vaccination protocol should be taken into account while interpreting the results. Other factors include the timing of sampling, presence and type of symptoms, productive and housing type, and presence (either suspected or confirmed) of other respiratory and immunosuppressive pathogens. 

The most suitable procedures for each of the main diagnostic purposes are described in Table 1.

## 5. Relevant Factors for IBV Control

The remarkable economic impact of IBV on poultry production encourages the implementation of massive vaccination strategies, whose wide adoption requires to settle between ideal application and practical aspects. As for other pathogens, IBV control can be eased by good management, correct bird density, air quality, all-in/all-out period duration, etc. [83]. Nevertheless, even optimally managed IBV-positive flocks were estimated to yield 3% less than IBV-free flocks [84,85]. 

The first essential barrier against IBV is biosecurity, with the strict implementation of external and internal measures regulating the flow of animals, people, supplies and waste/manure [86,87]. Adequate empty period and disinfecting procedures are necessary to reduce the risk of IBV infection dragging cycle after cycle. Farm density has been associated with a higher risk of viral transmission among farms; however, whethere this is due to airborne transmission eased by proximity or to the sharing of common risk factors (horizontal contacts or environmental conditions) remains to be established [88]. Nevertheless, biosecurity alone rarely guarantees the complete prevention of disease transmission.

To compensate for these aspects, vaccination is the most effective and frequently adopted option: even though it cannot fully prevent the infection, a decrease in clinical signs and infectious pressures can be achieved. For instance, H120-vaccinated animals showed a reduced viral transmission (R_0_ < 1) and shedding (more than 1 log_10_ of excreted titre reduction) after homologous challenge in experimental conditions [89]. 

Broiler vaccination relies on the administration of live attenuated vaccines, obtained from embryonated egg passaging of field strains [90]. These vaccines are commonly adopted for priming in layers and breeders, then inactivated vaccines can be administered for immunity boost [48,91], ensuring adequate maternal antibody transfer and protection of the reproductive tract [92,93]. On the other hand, inactivated vaccines are not as effective as live ones in inducing a proper protection and strong mucosal immunity at the tracheal level [94,95]. Hence, subsequent live attenuated vaccines can be administered between 4 and 6 weeks apart to enhance the protection in layers, depending on the infectious pressure [15]. Attenuated vaccines are most commonly used because of lower administration costs and the availability of mass administration procedures, both at hatchery and in the field. No individually administered vaccine would be suitable for frequent immunizations, thus limiting research on other types of vaccines, such as recombinant or subunit vaccines [90].

Nonetheless, the development of new vaccines based on reverse genetics [96] and recombination techniques is currently pursued. Although theoretically promising, the results of the available studies show partial protection against strains featured by a Spike gene closely related to the newly inserted one [97,98], but inadequate against heterologous ones [99]. Many of these genetic constructs [100,101] are based on a Beaudette strain (GI-1) backbone, which lacks of pathogenicity and would prevent residual effects of the attenuation process, others focus on the deletion of pathogenicity-associated genes [96], obtaining attenuation without the loss of protection. Recombinant vaccines have been produced also by cloning IBV proteins into the backbone of other viruses (Fowl Adenovirus, FAdV [102,103]; Newcastle disease virus, NDV [104,105]; avian Metapneumovirus, aMPV [106]), achieving a variable degree of protection. However, individual administration was still required for these vaccines, thus limiting their practicability. The choice of the insert region is currently the focus of intense research since it can deeply affect the achieved protection levels [98,99], because of innate protein immunogenicity and the contribution other factors such as conformational constraints, post-translational modifications or interactions with other proteins.

Other options, such as subunit vaccines [107,108,109], have also been explored, even though the absence of replication compared with attenuated vaccines constitutes a key limitation to the achievable protection, possibly requiring several and repeated administrations [90].

It is long-established that IBV extreme genetic variability impacts on the reliability of vaccine strategies in relation to the local epidemiological scenario and cross-protection among different strains [48]. Two schools of thought have often opposed each other on this topic, some in favour of homologous vaccination, others of heterologous one. This dichotomy is based on the principle that a homologous vaccine to the field strain is more likely to generate neutralizing immunity against it [90], whereas heterologous vaccination usually aims at providing a broader but less specific immunity towards different and potentially unknown circulating strains, in line with the “protectotype” concept [110,111,112,113]. Nevertheless, “hybrid” solutions are often applied, including one heterologous strain (e.g., Mass-based vaccine) and one homologous vaccine strain. 

Several vaccine combinations have been tested so far in line with the different epidemiological scenarios, with particular attention to Mass-like (GI-1) and 793B-like (GI-13) strains, American strains such as Ark (GI-9), Conn (GI-1), DE072 and GA98 (GIV-1), QX (GI-19), Q1 (GI-16) and Var2 (GI-23), suggesting a variable protection degree depending on the challenge virus [114,115,116,117,118]. The most common combinations, based on Mass-like and 793B-like strains, usually ensure good protection also against new variants [90,119]. A thorough evaluation of the most suitable and already available combinations should thus be performed before developing new variant-specific vaccines. 

Although the likelihood of cross-protection is slightly higher among more similar strains, there is a weak correlation between genetic similarity and protection of two strains [15], which hampers conclusions without experimental confirmation. In fact, the level of protection is usually evaluated by challenge infections of vaccinated animals, and the efficacy is assessed on clinical signs, challenge virus detection and ciliostasis, depending on the legal requirements dictated by Food and Drug Administration and/or European Pharmacopoeia [113]. This requires the involvement of animals, personnel, dedicated facilities and a great investment of resources and time, before reaching a reliable assessment of the cross-protection [120]. Serology is also of little help in establishing protection levels, since antibody levels and cross-reactivity lack correlation with protection [121,122]. New methods based on deep learning approaches have been proposed and could aid in predicting the protection levels between a vaccine and a new field strain, starting from the genetic and aminoacidic sequence of the S1 protein [123]. An approach free from clinical trial expenses and lengthiness would certainly help and speed up the response to new IBV strains appearing on the field.

Alternatively, an accessory evaluation of vaccine kinetics [68] and coverage [124,125] can be carried out by molecular assays (i.e., qRT-PCR), which quantify the vaccine titres replicating in the bird respiratory tract along the productive cycle. Because of the common persistence of the vaccines and the expected competition between vaccine and field strains for cellular receptors [68,82], the vaccine yield could be considered as a proxy of vaccination quality and protection, in addition to classical immunity assessment. Nevertheless, vaccination strategies in the field are often far from coherent, standardized or constant [74,126], even within the same country where the local epidemiological scenario is known. This heterogeneity hinders both the identification of field infections and the monitoring of vaccine intake. 

Besides vaccine type selection, the attempt of reducing labour and vaccine supply, inadequate mass administration, the use of fractional doses and wrong timing could significantly affect animal protection, coverage, vaccine-associated virulence and mixed vaccine-field virus circulation [46]. IBV vaccines are commonly administered by spray at the hatchery, by backpack sprayer or drinking water at the farm or, less frequently, by eye-drop application when attenuated, or by injection if killed [125,127]. The accuracy of the administration procedures deeply impacts on the achieved protection [15,125,128]. Vaccine route, dose and titre, temperature and quality of the water used for dilution, size of the sprayed particle, interactions with other vaccines, housing features such as flock size, ventilation and lights, are only some of the variables affecting vaccination efficacy [46,50,129] which need to be taken into account when planning a vaccine strategy.

Mass spray vaccination is an approach that clearly minimizes work whilst maximizing coverage, and it is usually applied at hatchery or farm by spray, which is considered analogous to the gold standard oculo-nasal vaccination in mimicking the entrance of respiratory viruses [120,130], when properly managed. However, improper settings of the diameter of the sprayed droplets can cause a deep penetration of the vaccine virus in the lungs and consequent vaccine reactions [120]. 

Spray vaccination could raise managerial problems when applied in a hatchery that supplies chicks to several farms requesting different vaccines. Nonetheless, the concern of chick contamination with different administered strains at the hatchery was diminished by Pellattiero et al., proposing that not yet replicating vaccines seem unable to spread to other non-vaccinated animals located in the same environment for several hours [131].

Spray vaccination has been progressively replacing administration via drinking water, which is largely deprecated since it often implies the combination of vaccines and other substances, normally dissolved in the water or to be co-administered, that could lower the vaccine titre or viability, leading to low coverage, rolling reactions and reversion to virulence. Nevertheless, it is still used, mainly for booster administration of less attenuated vaccines in the field [83]. Gel vaccination, initially studied for coccidiosis [132,133], has also been applied to IBV providing a chemically stable environment, without altering the vaccine kinetics [124,134]. A possible beneficial impact of gel vaccination on the chick body temperature with respect to the spray technique has also been hypothesized, although consistent experimental confirmation is still lacking. Vaccination timing is another field of intense debate and research. IB vaccination at hatchery is almost universally adopted, because of the higher coverage obtained while chicks are in the boxes. However, the ability of the birds to mount a proper immune response after an early priming is still questioned: experimental evidences suggest that the latter the birds are vaccinated, the highest antibody levels are reached [84,135], and the presence of maternally derived antibodies (MDA) could also complicate early vaccination by interfering with vaccine replication [136]. In field conditions, IB infection often occurs around 30 days of age [126,135]: this late appearance could be explained by the vaccine titre decrease and fading competition among replicating vaccines and field strains [68]. It is also worth to note that, when resorting to combined vaccination, an interval of at least two weeks between subsequent administrations appears beneficial likely because of a more mature immune system of the chick and a recovery of the respiratory epithelium [50,84]. 

On the other hand, many registered vaccines for broilers are declared to guarantee protection from day-old vaccination up to the end of the cycle, according to the supplementary protection certificates. An early combined vaccination at 1 day of age was proven to confer an efficient immune response [119], and epidemiological studies showed a lower likelihood of developing IB in flocks vaccinated with a combination of two strains at hatchery, probably because of a better standardization of vaccine administration conditions and procedures at the hatchery rather than at the farm [137]. Moreover, it should be considered that a delay in vaccination could expose the birds to an earlier entrance of the virus. Early vaccination appears therefore as a good compromise between bird biology and production constraints, ensuring fair conditions of vaccine administration, maximum coverage and economical sustainability of the procedures. 

It should not be under-emphasized that inconsistencies between experimental and field results could be due to different conditions and better control of environmental, managerial and biological variables in experimental settings [50], which prevent a full inference of the field conditions based on the obtained results. A certain delay was highlighted in the field response to a change of the vaccination strategy, demonstrating the existence of complex interactions among cross-protection between field and vaccine strains, infectious pressure and outbreak occurrence [126], stressing the need to wait at least two complete cycles in broiler production before assessing the actual effect of a particular vaccination strategy, especially in high infectious pressure situations.

As extensively described, IB control complexity is determined by several factors, from field conditions to the biological properties of the virus, which need unceasing research efforts and studies. New vaccines and new control strategies have to overcome old problems, such as costs and management issues, cross-protection limitations and variant emergence, whose knowledge and evaluation must be considered as essential requisites for strategy planning. 

At the moment, IB eradication is a distant goal and all efforts in the field should aim at optimizing the implementation of measures of well-known efficacy. A steady and rigorous monitoring based on objective criteria and knowledge of the local epidemiological scenario should be also strictly implemented. 

## Figures and Tables

**Table 1:** Diagnostic guidelines describing the suitable procedures based on the intended purpose.

Diagnostic Aim	Suitable Assays	Sampling Time	Suitable Samples
Confirmation of suspected infection (based on symptomatology)	Viral isolation ^a^	As soon as symptoms are observed, up to 10 days post-infection	Well-preserved individual or pooled samples; tracheal specimens are preferable; kidneys and oviduct may be sampled based on symptoms
	Molecular assays ^b^: RT-PCR, qRT-PCR	As soon as symptoms are observed	Individual or pooled samples; tracheal/cloacal specimens; kidneys and oviduct may be sampled based on symptoms
	Serology: ELISA, VN ^c^, HI ^c^, AGP	From 7 days post-infection (2 weeks for ideal results)	Sera
Surveillance (absence of infection)	Molecular assays ^b^: RT-PCR, qRT-PCR	Anytime; if vaccination is implemented, infection occurrence is more probable when vaccines start to fade	Individual or pooled samples; tracheal/cloacal specimens
	Serology: ELISA		Sera
Immunological response to vaccination	Serology: ELISA, VN ^c^, HI ^c^	From 7 days post-vaccination onwards; longitudinal studies may be performed for more informative results	Sera
Vaccination coverage	Molecular assays ^b^: RT-PCR, qRT-PCR		Individual samples; tracheal/cloacal specimens
Vaccination kinetics (replication and persistence)	Molecular assays ^b^: qRT-PCR		Individual or pooled samples; tracheal/cloacal specimens

^a^ IBV presence must be confirmed by other techniques. ^b^ Do not assess viral viability; coexistent strains may be discriminated by genotypization or using specific assays. ^c^ Allows for serotypization.
